# Rheological Properties, Textural Properties and Storage Stability of Sauce Enriched with Pomace from Oxheart Tomatoes (*Lycopersicon esculentum*)

**DOI:** 10.3390/foods14152627

**Published:** 2025-07-26

**Authors:** Dumitrița Flaiș, Mircea Oroian

**Affiliations:** 1 Faculty of Food Engineering, “Ştefan cel Mare” University of Suceava, 13th University Street, 720229 Suceava, Romania; dumitritaflais96@gmail.com; 2 Suceava-Botoșani Regional Innovative Bioeconomy Cluster Association, 720229 Suceava, Romania

**Keywords:** sauce, physicochemical parameters, textural properties, oxheart tomatoes

## Abstract

The objective of this study was to develop a novel sauce formulation in which egg yolk was substituted with pea and soy proteins, in addition to the incorporation of tomato pomace as a functional ingredient. Nine experimental samples (E1–E3, S1–S3, and P1–P3) and three control samples (E0, S0, and P0) were prepared, corresponding to three protein sources (E: egg yolk, S: soy, P: pea), with increasing concentrations of tomato pomace (0, 2, 4, and 6%). The formulations were adjusted proportionally in terms of water and oil to maintain the desired consistency. The analyses performed included: physico-chemical analysis of the sauce (fat content, peroxide value, and CIE L* a* b* color determination), quality assessment using Fourier Transform Infrared Spectroscopy (FT-IR, rheological measurements, and microstructural evaluation. The sample designated P2 demonstrated a notable correlation with favourable parameters, exhibiting intense colouration, elevated protein content, and consistent rheological properties. However, at higher levels of tomato pomace (notably 6%), microstructural instability was observed, which may limit the formulation’s robustness over time. These findings demonstrate that tomato pomace can enhance the functional and structural characteristics of sauce, while also highlighting the importance of optimizing concentration levels to avoid negative impacts on emulsion stability. Overall, the results support the use of tomato pomace and plant proteins in the formulation of sustainable and innovative food products.

## 1. Introduction

Tomato pomace (TP), a by-product of the tomato processing industry, is produced in substantial quantities but is frequently underexploited, contributing to resource inefficiency and environmental degradation [1]. It typically accounts for 5–30% of total production output and consists primarily of tomato peels, seeds, and fibrous residues [2]. When subject to appropriate processing and utilization strategies, TP holds potential for notable economic return [3]. This by-product is rich in bioactive compounds such as lycopene, carotenoids, flavonoids, essential vitamins, and soluble dietary fiber (SDF), all of which are linked to important nutritional and functional properties [3,4,5]. Despite its demonstrated benefits in bakery, meat analogues, and snacks, its use in emulsified sauces such as sauce remains largely unexplored—representing a research gap this study seeks to address.

In addition, TP offers a considerable protein profile, comprising approximately 21.9% protein in raw pomace and up to 38.7% in defatted tomato seeds [6], making it a nutritionally relevant component for animal feed [7]. Research has indicated that including TP in animal diets can deliver various benefits, including increased α-tocopherol content, better oxidative stability of meat, reduced feed costs, and enhanced milk quality [8,9].

These findings suggest that the bioavailability and digestibility of TP’s nutrients and antioxidants support oxidative defense and contribute to improved quality of animal-derived products [10]. Translating these functional advantages into plant-based emulsified systems may offer a dual benefit: valorizing an agro-industrial waste and improving product performance.

Leguminous plants, of which the pea is an example, have been cultivated for millennia on account of their high protein content and nutritional value. They are rich in protein, fibre, vitamins and minerals, and are used in many forms, including flour, starch, and protein isolate. In the contemporary food industry, pea ingredients are highly regarded for their versatility, as evidenced by their capacity to substitute for eggs in baked goods, function as emulsifiers, texturisers or thickeners in soups, sauces, snacks, and meat products. Pea ingredients are characterised by their functional properties and nutritional value, which contributes to their ease of digestion, low anti-nutritional factor content, ability to regulate blood glucose levels, and support for digestive health. Consequently, pea ingredients are regarded as a sustainable and promising source of plant protein for the global food industry [11].

At present, the most widely utilised sources of textured vegetable protein are those derived from soy, peanut, pea, and wheat gluten [12]. Research in this field has demonstrated that the selection of raw material has a substantial impact on the final characteristics of textured proteins. For instance, in the context of soy protein, the extrusion process instigates significant physico-chemical alterations that result in a distinct structure, accompanied by enhanced functional properties [13].

The textured vegetable proteins developed in this manner possess a fibrous-stratified internal organisation with an orderly oriented structure. This influences both their digestibility and their degree of absorption, as well as their technological behaviour. Soy protein is regarded as a highly promising candidate for use as a meat substitute due to its gel-forming ability, balanced nutritional profile, and low cost [14]. While both soy and pea proteins have proven emulsifying capacity and are widely used in dairy and meat analogues, their performance in oil-in-water emulsions such as sauce—especially in the presence of fiber-rich compounds like tomato pomace—requires further elucidation.

The protein sources were selected for their emulsifying capacity and nutritional value, considering differences in purity and functional performance. Vegetable soy and peas have been identified as excellent sources of protein for sauce due to their ability to emulsify effectively, thereby providing a creamy texture and stability to the product. Consequently, they can be utilised as effective replacements for egg in vegan and allergen-free recipes.

In light of the increasing demand for sustainable, functional food formulations, this study explores the development of a tomato-based sauce enriched with tomato pomace (TP) and formulated with different protein sources. Tomato pomace, rich in bioactive compounds such as lycopene and dietary fiber, represents a valuable ingredient with both nutritional and technological benefits.

To investigate its potential as a functional food component, three sauce prototypes were developed using distinct protein emulsifiers: egg yolk, soy protein, and pea protein. These ingredients were selected for their emulsifying properties, nutritional profiles, and relevance in plant-based food design. This multifactorial study addresses a gap in the literature regarding the combined use of agro-industrial by-products and alternative proteins in emulsified systems. The study aims to evaluate how different protein sources influence the physico-chemical, rheological, and sensory properties—and overall stability—of tomato-based emulsified sauces. To facilitate interpretation, comparisons with mayonnaise are included throughout the study. Although the final product is referred to as a sauce, the formulation is nearly identical to that of conventional mayonnaise, with quantitative differences in specific ingredients. Therefore, established analytical frameworks and benchmarks from mayonnaise research were considered applicable and relevant to the present investigation.

## 2. Materials and Methods

### 2.1. Materials

The following materials were used to make sauce: yolk powder, pea protein, soy protein, water, oil, vinegar, salt, and mustard.

### 2.2. Methods

#### 2.2.1. The Emulsification Process

The oil, vinegar, and mustard were procured from a local supermarket, while the yolk powder, pea protein, and soy protein were sourced from a local grocer. Tomato pomace was prepared from the peel and seeds of oxheart tomatoes obtained from a local producer. The aqueous phase was composed of water and vinegar. Prior to incorporation into the sauce, the protein ingredients underwent a hydration process and were left to stand at 4 °C overnight. The base mixture was subjected to homogenisation at 2000 rpm. Thereafter, the tomato pomace (0–6%) and sunflower oil were added in a gradual manner, contingent upon the specific formulation. The final homogenisation stage was conducted for a duration of 2.5 min, after which the samples were stored at a temperature of 4 °C. In order to maintain comparable consistency across samples, the sunflower oil content was progressively reduced and compensated by increasing the water content. The quantities of mustard, salt, and vinegar were kept relatively constant across formulations, with only slight adjustments to maintain balance in taste and stability. However, it is acknowledged that the resulting emulsions differ in overall composition due to the variation in the oil-to-water ratio. Consequently, observed variations in physico-chemical and sensory characteristics may be indicative of a combined effect of protein type, tomato pomace level, and emulsion composition. These adjustments enabled the investigation of the effect of tomato pomace and protein source (egg yolk-E, soy-S, or pea protein-P) on the product’s structural and functional properties to be conducted in isolation. Table 1 presents the detailed formulation compositions.

#### 2.2.2. Physico-Chemical Analysis of Sauce

##### Fat Content

The fat content was determined using the Soxhlet method with an automatic analyzer type SER 148/6 (VELP, Milan, Italy). For this analysis, 5 g of sauce sample were weighed, placed in a porous thimble, and subsequently extracted with petroleum ether as solvent. The protocol of the Soxhlet apparatus included the following steps: a 60-min immersion phase, followed by a 10-min removal step, then a 60-min washing step, solvent recovery for 20 min, and finally a 5-min cooling period. After the completion of the extraction process, the collection cuvettes were placed in an oven at 105 °C for a period of one hour, with the objective of drying them. The collection cuvettes were then transferred to a desiccator, where they were allowed to cool. Once cooled, the cuvettes were weighed, and the percentage of fat was calculated based on the mass difference [15]. Although this method is widely used, it should be noted that applying Soxhlet extraction directly to high-moisture emulsified samples (such as sauce), without a preliminary dehydration or hydrolysis step, may lead to a slight underestimation of the total fat content. This potential limitation was considered during data interpretation.

##### Determination of Protein Content (Kjeldahl Method)

The protein sources used in the formulations included egg yolk powder (approx. 32% protein), soy protein isolate (min. 90% protein), and pea protein concentrate (approx. 80% protein), based on values declared by the respective manufacturers. These differences in protein concentration and functional properties were considered when interpreting the final protein content results of the emulsified sauces. The protein content of the sauce samples was determined using the classical Kjeldahl method. Approximately 1 g of each sample was weighed and digested with 15 mL of concentrated sulfuric acid (H_2_SO_4_) in the presence of a catalyst mixture (typically potassium sulfate and copper or selenium). The digestion was carried out until a clear solution was obtained, indicating complete conversion of organic nitrogen to ammonium sulfate. After cooling, the digested mixture was neutralized with 40% sodium hydroxide (NaOH) and distilled using a Kjeldahl distillation unit. The liberated ammonia was collected in a boric acid solution (2%) and titrated with standardized 0.1 N hydrochloric acid (HCl) until the endpoint was reached. The percentage of nitrogen was calculated, and the total protein content was obtained by multiplying the nitrogen value by a conversion factor of 6.25, which is appropriate for mixed protein sources. All measurements were carried out in duplicate, and the results are expressed as mean ± standard deviation [16].

##### Peroxide Value

To ascertain the peroxide value, the 12 samples were prepared in advance. In each of the nine samples and three control samples, 1 g of product was taken. The samples were then subjected to a series of steps to facilitate the extraction of fat. To this end, 10 mL of chloroform were added individually, and the samples were shaken until the fat had dissolved. However, no physical separation (e.g., decantation or centrifugation) of the fat phase was performed. The analysis was carried out directly on the emulsified system. Thereafter, 15 mL of glacial acetic acid and 1 mL of potassium iodide were added. The samples were then kept in the dark for a period of five minutes. Following this, 1 mL of starch was added, and the samples were titrated until the yellow colour disappeared. All emulsified samples were stored in sealed containers at 4 ± 1 °C, protected from light, and the peroxide value was determined 24 h after emulsification. All measurements were performed under identical storage and analytical conditions to ensure the reliability of comparative data. The peroxide value was expressed in meq O_2_/kg, calculated using the following Formula (1) [17]:

(1) $$\mathrm{IP}=\frac{\left(V-v\right)*F*0.00127*100}{G}$$

where:

*V* = mL of 0.01 N sodium thiosulfate solution consumed when titrating the fat sample;

*v* = mL of 0.01 N sodium thiosulfate solution consumed for control;

*F* = correction factor for the sodium thiosulfate solution;

0.00127 = the amount of iodine (g) equivalent to 1 mL of 0.01 N thiosulfate solution;

*G* = weight of the fat analyzed (g).

##### CIEL* a* b* Determination

The colour was determined by means of a CM-5 spectrophotometer (Konica Minolta, Foster City, CA, USA). This analysis is of significance as it demonstrates that the introduction of pomace from tomatoes in varying amounts results in the tomatoes acquiring a brick-red colour, whilst concomitantly conferring certain health benefits as a potent antioxidant. The colour was determined using a method described by Stinco, with some modifications [18]. A 5 g sample was obtained and transferred into a transparent container. The CIE scale L* a* b* was employed for analysis, with three replicates considered for each sample. Colour intensity (chroma) was calculated using Equation (2):

(2) $$C^{*}=\sqrt{a^{{*}^{2}}+b^{{*}^{2}}}$$

where:

$C^{*}$—is chroma (colour intensity),

a*—is the red-green coordinate,

b*—is the yellow-blue coordinate.

The total colour difference between two samples (ΔE*) was calculated using Equation (3):

(3) $$∆E^{*}=\sqrt{{∆L}^{{*}^{2}}+{∆a}^{{*}^{2}}}+{∆b}^{{*}^{2}}$$

where:

$∆E^{*}$—is the perceived difference in colour, and ΔL*, Δa*, and Δb* are the differences between the corresponding L*, a*, and b* values of the two samples.

Thixotropic behaviour was assessed using a decay model, as shown in Equation (4):

(4) $$\eta \left(t\right)=\eta_{0}*e^{-kt}$$

where:

$\eta \left(t\right)$—is the viscosity at time t,

$\eta_{0}*$—is the initial viscosity,

k—is the thixotropic constant,

t—is the time under shear.

##### Quality Assessment of Sauce by FT-IR Spectroscopy

The absorption spectra of the freeze-dried samples were obtained by means of Fourier transform infrared spectroscopy (FTIR) (Nicolet iS20, ThermoScientific Inc., USA), equipped with an attenuated total reflectance (ATR) accessory. The samples were then brought into direct contact with the ZnSe crystal by applying pressure to ensure proper contact. Spectral data were collected in the range of 4000–550 cm^−1^ at room temperature. The obtained spectra were analysed using Spectrum™ 10 software (Thermo Fisher Scientific Inc., Waltham, MA, USA).

#### 2.2.3. Rheological Properties

##### Steady State Rheology

Thixotropy analysis of sauce was performed using a Mars 40 rheometer (Thermo Haake, Germany) equipped with a system of parallel plates 40 mm in diameter with a working distance of 1000 μm between the plates. Prior to testing, each sample was allowed to stand for 5 min to allow the internal structure to recover and reach the test temperature. All rheological measurements were performed at a constant temperature of 20 °C. The shear rate (γ, s^−1^) was varied from 0 to 100 and back to 0 s^−1^, while the corresponding values of shear stress (τ, Pa) and dynamic viscosity (η, Pa·s) were recorded. Each determination was carried out in triplicate to ensure reproducibility of the results [19].

The hysteresis area between the ramp up and the ramp down curves was calculated to quantify the thixotropic behavior of the material.

##### Viscoelastical Properties

In addition to the determination of thixotropy, other parameters essential for characterising the viscoelastic behaviour of the samples such as storage modulus (G′), loss modulus (G″), complex modulus (|G*|) and complex viscosity (|η*|) were evaluated in the rheological analysis. These indicators provide valuable information on the internal structure, stability and consistency of the tested systems, contributing to a broader understanding of the behaviour of plant protein sauce under dynamic stress. The stress sweeps were set at 1 Hz for determining the viscoelastic region.

#### 2.2.4. Microstructure of Sauce

Optical microstructure analysis was performed using a Stereomicroscop Zeiss Primostar 3 with color camera AxioCam 208 (Carl Zeiss IMT Co., Ltd., Shanghai, China) and its software, Zen, ver. 3.4.91.00000. Observations were performed at 40× magnification after each sample was diluted with 50 µL of distilled (DI) water to reduce the density of the system and facilitate visualization of the internal structure. The diluted sample was applied to a glass slide, then covered with a thin coverslip to ensure the formation of a uniform film and to prevent optical interference caused by air bubbles. This dilution step was necessary to avoid overlapping of droplets and to allow clearer optical resolution during stereomicroscopy. However, it is acknowledged that the addition of water may result in a slight alteration to the size or spacing of dispersed droplets. Accordingly, the analysis was interpreted in a qualitative and comparative manner across all samples, with identical preparation conditions employed in order to preserve consistency. This methodology enabled a qualitative assessment of the distribution and size of the dispersed phase droplets, the identification of any agglomeration or coalescence, and the homogeneity of the system. Consequently, microstructural analysis offers supplementary pertinent data concerning the physical stability of emulsions and the impact of composition on phase organisation.

#### 2.2.5. Analysis of Organoleptic Properties

The sensory evaluation of three types of sauce from the above samples was carried out using Quantitative Descriptive Analysis (QDA). 20 volunteers aged between 18 and 65 years participated in the study. Prior to the evaluation, the panellists were instructed in the sensory vocabulary and rating scale used in a short familiarisation session to ensure a common understanding of the evaluation criteria.

The samples were randomly coded with three digits and presented in a randomised order to minimise the influence of positioning on perception. The tasting was conducted at room temperature, using fresh toast and plain biscuits as food carriers, and participants were provided with drinking water between samples to neutralise taste. The following sensory attributes were analysed: appearance, flavour, mouthfeel, mouth consistency, mouthfeel of fat and overall acceptability. The ratings were made on a scale from 1 (minimum intensity) to 9 (maximum intensity). The scores were then normalised to the average given by each rater for each attribute and recalibrated using the calibration curve derived from the ratio of the raw scores to the normalised scores. This method corrected for individual differences in scale use. The results were expressed as mean values for each attribute, corresponding to each type of sauce analysed.

#### 2.2.6. Statistical Analysis

Principal component analysis (PCA) was performed using Unscrambler X 10.1 (CAMO, OsloNorway), while one-way analysis of variance (ANOVA) was conducted using the XLSTAT (trial version) software. A significance level of *p* < 0.05 was applied to all tests.

## 3. Results and Discussion

### 3.1. Physico-Chemical Analyses

The determination of the peroxide value is an essential method for assessing the quality of fats and oils as it provides information on the degree of primary oxidation. High concentrations of peroxides indicate the presence of ongoing oxidative processes that may adversely affect the stability of the product and lead to rancid taste and odour. The analysis of the samples showed a significant variation in the peroxide values between the different samples. Sample E0 had the lowest value (1.67 meq O_2_/kg), indicating a low level of oxidation and good stability at the time of analysis. Samples from this control (E1–E3) showed slightly higher values, ranging from 2.2 to 3.02 meq O_2_/kg, which could indicate moderate exposure to oxidation-promoting factors (such as oxygen, light, or temperature). In contrast, S0 exhibited a peroxide value of 3.75 meq O_2_/kg, which was significantly higher than that of the control group (E), indicating a more pronounced progression of oxidative processes. However, the derived samples (S1–S3) exhibited lower values, ranging from 2.04 to 2.67 meq O_2_/kg, an unusual situation that could be attributed to factors such as differences in storage conditions, potential antioxidant treatment, or possible experimental variations. P0 exhibited an intermediate value of 3.02 meq O_2_/kg; however, sample P1 reached a peroxide level of 5.27 meq O_2_/kg, suggesting the occurrence of an advanced oxidation process, potentially attributable to unfavourable handling or storage conditions. The values of the other two samples in this group (P2—3.47 meq O_2_/kg and P3—2.2 meq O_2_/kg) support the hypothesis of significant variability in the stability of these samples. It is noteworthy that the peroxide values were determined directly on the emulsified samples without prior separation of the lipid phase. Therefore, the presence of water, proteins, and emulsifying agents may have led to an underestimation of the absolute peroxide values. Nevertheless, as all samples were subjected to identical experimental conditions, the relative differences observed are considered valid and provide reliable comparative information on the oxidative stability of the formulations. It should be noted that the experimental design involved simultaneous variation of three compositional factors: the type of protein, the concentration of tomato pomace, and the oil-to-water ratio. These parameters are known to influence emulsion stability and oxidative processes in a synergistic manner. Consequently, the observed differences in peroxide values cannot be attributed to any single factor independently. This combined effect represents a methodological limitation, which is acknowledged in this study. Future work will consider a more controlled experimental approach, in which the oil-to-water ratio is kept constant, allowing clearer interpretation of the individual impact of protein type and pomace concentration. ANOVA results confirmed significant differences in peroxide values among the samples across all groups, as indicated by high F-values (e.g., 670.36 for group P) and *p-values* < 0.05, validating the variability in oxidative stability. Peroxide value measurements revealed significant differences between samples. These findings support the hypothesis that oxidative stability is closely linked to both the type of protein used and the presence of tomato pomace, reflecting their compositional impact on lipid oxidation resistance. In general, lower values indicate a satisfactory state of preservation, while higher values suggest a greater risk of oxidative degradation. These findings provide a robust foundation for evaluating the risk of rancidity and for determining optimal processing and storage conditions. Although peroxide value primarily reflects oxidative stability, it can also be indirectly affected by the physical stability of emulsion. Larger droplet sizes or reduced viscosity may facilitate lipid coalescence and increase surface exposure to oxygen, thus accelerating oxidation processes.

Beyond formulation composition, several technological and natural strategies have been investigated to enhance both the physical and oxidative stability of sauce, offering relevant insights for the development of improved formulations. From a physico-chemical perspective, emulsion stability is influenced by factors, such as phase density difference, droplet size, and the viscosity of the emulsion, as demonstrated by Dhiman et al. [20]. In accordance with the stipulations set out in Stokes’ law, a decline in emulsion stability is to be expected in the event of a decrease in viscosity and an increase in droplet size. Furthermore, Tavakoli et al. reported that ultrasound treatment improves the storage stability of mayonnaise emulsions, particularly when combined with thickeners such as xanthan gum (XG), guar gum (GG), and XG/GG mixtures—achieving better performance than commercial samples.

In parallel, other studies have highlighted the role of bioactive compounds in improving oxidative stability. Several studies have demonstrated the efficacy of natural extracts in enhancing the oxidative stability of mayonnaise. As demonstrated by Kishk et al., the incorporation of ginger powder (0–1.25%) significantly enhances the antioxidant activity of mayonnaise [18]. In addition, Li et al. reported that anthocyanin-rich extracts from purple maize enhance antioxidant performance during storage. Similarly, Rasmy et al. observed analogous effects subsequent to the incorporation of an ethanolic sage extract [21].

Similarly, Rasmy et al. observed analogous effects after the incorporation of ethanolic sage extract. Martillanes et al. [22] and further confirmed the enhanced antioxidant activity of fenugreek seed extracts (FSE) over leaf extracts (FLE), attributing this to the higher phenolic content of the seeds, encompassing flavonoids, saponins, and alkaloids [22]. The substitution of egg yolk with corn starch in mayonnaise formulations resulted in a noticeable reduction in fat content, with reported values of 41.45% for a sample containing 5% egg and 0% starch, and 40.32% for a formulation with 0.5% starch and no egg. These findings support the observation in the present study, where variations in fat content were significantly influenced by the type of protein used and the addition of tomato pomace [23]. The present analysis demonstrated significant variations in fat content depending on the protein source and the addition of tomato pomace. Control samples (E0) exhibited the highest fat content (~32%), whereas formulations containing pea protein (P group) showed the lowest fat content (as low as ~12%), indicating decreased lipid retention due to compositional factors. Soy protein samples (S group) displayed intermediate fat levels (~12–14%), with observable variability among replicates. Similarly, peroxide values and protein contents differed significantly among the groups, reflecting the influence of these ingredients on oxidative stability and nutritional composition. These findings highlight the critical role of protein source selection and functional additives in optimizing the physicochemical and sensory attributes of sauce formulations. The fat content exhibited significant differences between samples within groups, evidenced by high F-values (e.g., 2188.46 for group P) and *p-*values below 0.05, suggesting fat levels were statistically different across formulations.

The protein content measured across the three groups of samples (E, S, and P) exhibited biologically plausible values, although with differing degrees of variability between groups. Group E (egg yolk) presented an average protein content around 7–10%, with relatively low standard deviations (0.15–0.20), indicating good repeatability and homogeneity within these samples. The ANOVA results for protein content in this group showed a highly significant F-value of 284.19 (*p* < 0.0001), demonstrating that there are statistically significant differences among the samples in this group. In group S (soy protein), the protein content ranged from approximately 4.0% to 7.6%, with moderate variability (standard deviations between 0.20 and 0.30). The ANOVA F-value of 28.43 (*p* < 0.000) indicates significant differences between the soy-based samples, confirming that variations in protein content are statistically meaningful.

Group P (pea protein) exhibited the highest average protein contents, ranging from approximately 3.8% up to 5.8%, accompanied by slightly higher variation (standard deviations up to 0.25). The F-value of 55.71 (*p* < 0.0001) confirms significant differences among these samples as well, although the magnitude of variation is lower compared to the E and S groups. Overall, the significant F-values across all groups indicate that the differences in protein content between the respective samples are statistically significant (*p* < 0.05). These results align with the compositional differences expected from the formulations and confirm the reliability of the protein quantification method used.

A comparative perspective can be drawn with the study by Park et al., who developed low-fat mayonnaise-type emulsions containing 15–30% oil using spirulina and soy protein isolate. Their formulations showed acceptable structural and oxidative stability within this reduced-fat range [24].

In the present study, the formulations based on pea protein and tomato pomace (P0–P3) demonstrated even lower fat levels, ranging from 11.89% to 20.12%. Despite the substantial reduction in lipid content, these samples maintained good emulsion integrity and oxidative stability, as shown by both the peroxide value and rheological results.

These findings confirm the functional potential of combining legume-derived proteins with fiber-rich plant residues as structuring agents in reduced-fat emulsions. The observed differences were statistically significant (F-value = 2188.46, *p* < 0.05), highlighting the strong influence of formulation composition on fat retention. Compared to the emulsions developed by Park et al., the present study achieved further reductions in fat content without compromising physicochemical or functional performance, strengthening the case for clean-label, plant-based alternatives in mayonnaise-type products [25].

The study conducted by Tavakoli et al. investigated the effect of different types of vegetable oil on the protein content of mayonnaise, with chicken egg yolk as the primary protein source, which contains approximately 15–16% protein [15]. In all formulations, the amount of yolk was constant, so that differences in the final protein content were due exclusively to variation in the composition of the oils used. Analysis of variance demonstrated statistically significant differences (*p* < 0.05) between treatments. The range of protein content values was from 1.61 to 1.98%, all of which met the minimum quality requirements of the Indonesian standard SNI 01-4473-1998 (minimum 0.9% protein). The highest protein content was observed in the coconut oil formula (P2—1.98%), followed by P1 (1.90%), P3 (1.84%) and the lowest value was recorded in sunflower oil (P4—1.61%) [15].

The incorporation of tomato pomace led to a gradual decrease in brightness and an increase in colour intensity (higher values of a*, b* and C*), indicating the escalating visual impact of the plant extract (see Table 2). The M (pea) samples exhibited the most intense colours, as indicated by the highest a*, b*, C*, and ΔE* values, suggesting a more pronounced and distinct colour compared to the reference sample. The S (soybean) samples are at an intermediate level, with a visible but less intense colour than the pea samples. The G (yolk) samples exhibited less intense colours than the other groups, maintaining a shade closer to the classic sauce. The influence of tomato pomace on the colour of sauce is significant, with this effect being more pronounced at higher amounts. The type of egg replacer used also had a significant impact on the colour of the sauce, with peas enhancing the colour the most, followed by soybeans and finally yolk. These parameters can be utilised to assess the visual appearance of the products and to adapt the recipe according to consumer preferences. ANOVA analysis confirmed these differences were statistically significant, with very high F-values observed for L* (1315.94), a* (1279.06), b* (3276.69), and C* (7607.66), all with *p*-values < 0.05. The total colour difference (ΔE*), which quantifies the overall colour change relative to the control sample, also increased with tomato pomace concentration, reaching values of 26.88 ± 0.21 in pea protein samples (P2), indicative of a perceptible and significant colour modification. While the type of protein and tomato pomace concentration clearly influenced the colour profile, other compositional variables such as fat and water content, as well as the degree of fat dispersion in the aqueous phase, may also have contributed to the observed differences. The physical structure of the emulsions can affect light scattering and thus influence the visual appearance of the final product. These results demonstrate that both the amount of tomato pomace and the type of protein source significantly affect the colour characteristics of sauce formulations, which can be quantitatively assessed and tailored according to consumer preferences.

As proposed by X. Liu et al., the colour of mayonnaise, an essential sensory attribute influencing consumer perception and aesthetic appeal of the product, is affected by its composition (particularly egg yolk and corn oil, which are rich in fat-soluble carotenoids) as well as the heat treatments applied during the technological process [26]. As demonstrated in the findings presented by L. Liu et al., a gradual decline in the lightness (L*), redness (a*) and yellowness (b*) values was observed in the control (CM) samples as the pasteurisation temperature increased from 68 to 76 °C [18]. The present study hypothesizes that the application of more intense heat treatments results in a darker colouration of mayonnaise, a phenomenon that may be attributable to the thermal aggregation of LEY (lipid complex in yolk), which has been shown to reduce dispersion efficiency at the oil-water interface. This assertion is supported by the findings of Li et al. [27]. In comparison with the control mayonnaise (CM), samples that had undergone treatment with betaine (BM) and proline (PM) exhibited variations in their colour behaviour. In general, BM and PM appeared to contribute to maintaining a more stable colour, with brightness and colour saturation values similar to those observed in pasteurised samples treated at lower temperatures. This observation may be attributed to interactions between amino acids and LEY, which have been shown to slow thermal aggregation of proteins and facilitate better dispersion of oil droplets [28].

The ΔE* values, obtained by comparing each sample to its respective control, ranged from 0 (E0, S0, P0) to 26.88 ± 0.21 (P2). The relatively low colour differences observed in the E (egg yolk) and S (soybean) groups indicate moderate changes in chromatic parameters, suggesting partial yet controlled extraction of carotenoids. In contrast, the higher ΔE* values in the P (pea protein) group, particularly for samples P2 and P3, reflect a more intense extraction of colour compounds, likely due to better compatibility between rapeseed oil and lipophilic pigments. These findings are favourable, highlighting both the colour stability of certain extracts and the high efficiency of rapeseed oil as a green solvent for carotenoid recovery.

### 3.2. FT-IR Spectroscopic Evaluation of Emulsified Sauce Samples

**Figure 1:**
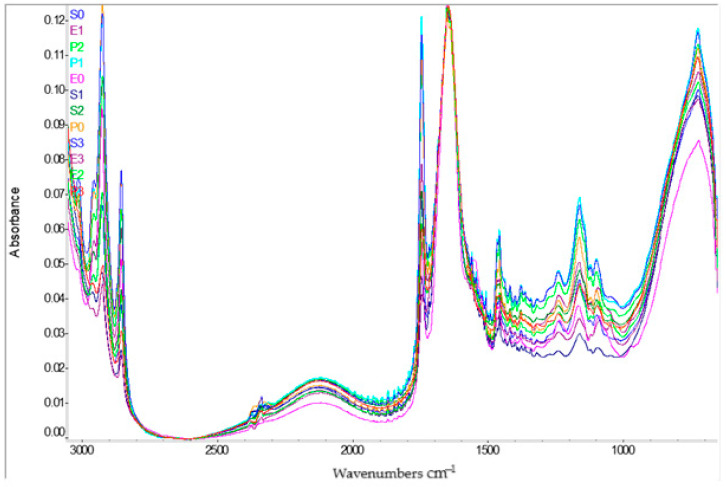
FT-IR spectra of emulsified sauce samples formulated with different protein sources (egg yolk, soy, and pea).

Figure 1 illustrates the superimposed FT-IR spectra of the sauce samples, identified in the legend by their formulation codes (e.g., E0–E3 for egg-based, S0–S3 for soy-based, and P0–P3 for pea-based samples). Spectral data were acquired in the mid-infrared range to highlight functional groups relevant to the chemical structure of the emulsions.

A broad and intense absorption band around 3300 cm^−1^, is observed across most samples, corresponding to O–H stretching vibrations, indicative of hydroxyl groups present in phenolic compounds, alcohols, or water. Another distinct region around 2900 cm^−1^ is associated with C–H stretching vibrations, characteristic of methyl and methylene groups found in aliphatic chains. A pronounced signal is also observed at around 1600 cm^−1^, which can be attributed to C=C stretching vibrations in the aromatic structure or C=O (carbonyl groups), as found in polyphenols or flavonoids. Between 1000–1300 cm^−1^, the presence of C–O stretching vibrations is indicated, suggesting the existence of alcohols, esters or carboxylic acids. The spectra of the various samples demonstrate a comparable profile, indicating the presence of shared functional compounds within the samples. However, minor variations in the intensity and precise position of the absorption bands are discernible, indicating compositional differences inherent to each plant or the specific treatment applied. A thorough analysis of the FT-IR spectra reveals significant variations between the analysed samples, particularly within specific regions of the frequency range. In the range of 1600–1700 cm^−1^, significant variations in the intensity of the bands are observed, with a more pronounced absorption in the case of M1 (red curve), which may indicate a higher concentration of compounds containing carbonyl groups (C=O) in or structured C=C double encounters, aromatic frequencies.

A further area in which discernible disparities become evident is that between 1030 and 1150 cm^−1^, where the spectra of pea-based samples exhibit more intense bands. These differences can be attributed to the presence of C–O groups, which are characteristic of alcohols, esters, and carbohydrates. Furthermore, within the region of 2800–3000 cm^−1^, corresponding to the stretching vibrations of the C–H bonds in aliphatic chains, slight intensity variations are observed. These variations indicate differences lipid content between the samples. P1 is distinguished by higher intensities in several regions of the spectrum, especially around 1740 cm^−1^ and in the area around 1050 cm^−1^. These characteristics are indicative of a higher concentration of esters and fatty acids, which may signify a more abundant phytochemical composition. Conversely, the soybean samples exhibited more uniform spectra, characterised by bands of reduced intensity in specific regions. This finding suggests a more balanced composition or a lower concentration of aromatic or reactive compounds.

In the FT-IR spectrum of sample P2, an intense band is observed at 1720 cm^−1^, corresponding to the C=O vibration. This is absent or weak in the other samples, suggesting efficient functionalisation with carbonyl groups. Furthermore, the observed shift of the OH band from 3420 cm^−1^ to 3360 cm^−1^ is indicative of the formation of hydrogen bonds, a process which contributes to structural stability and favourable molecular interactions. These characteristics lend support to the hypothesis that P2 is the optimal formulation.

Daoud et al. identified the peak corresponding to the H–O–H band in water molecules, located around 1650 cm^−1^ [29]. FT-IR spectrum of soy protein isolate (SPI) revealed a specific peak around 3300 cm^−1^, corresponding to the stretching vibrations of the hydroxyl (–OH) groups of amino acids, as observed by Daoud et al. [29]. Furthermore, characteristic bands at 2980 cm^−1^ and 1400 cm^−1^, associated with stretching vibrations of carbon–hydrogen (C–H) bonds, were identified. The presence of bands at 1700 cm^−1^, 1500 cm^−1^ and 1360 cm^−1^ has been shown to be specific to amide I, amide II and amide III [30]. These bands are indicative of the protein structure through vibrations of carbonyl groups (C=O), hydrogen bonds (N–H) and carbon–nitrogen bonds (C–N). In addition, Daoud et al. identified the peak corresponding to the H–O–H band in water molecules, located around 1650 cm^−1^ [31]. The spectrum of pectin (PEC—pure pectin) exhibited intense peaks at 1025 cm^−1^ and 960 cm^−1^, attributed to the stretching vibrations of the carboxylate (COO–) and ether (C–O) groups, as well as the ether-ether (C–O–C) group, which are typical of this polysaccharide. In the case of maltodextrin (MD—a polymer obtained by partial hydrolysis of starch), the spectrum exhibited significant bands at 3400 cm^−1^ (–OH) and 3000 cm^−1^ (C–H), which are specific to the functional groups present within the polysaccharide structure. Furthermore, an intense peak was identified at 1800 cm^−1^, corresponding to the methyl ester group (COOCH_3_), and another at 1560 cm^−1^, associated with the asymmetric stretching vibration of the carboxylate (COO^−^) group [32,33,34]. The spectrum of the vegetable oil, which is rich in fatty acids (referred to as High-Weighted Oil—HWO), was analysed in free (unencapsulated) form. This analysis revealed characteristic absorption bands at 3000 cm^−1^ (–OH groups) and 2930 cm^−1^, which are attributed to the unsaturated aliphatic structure (C=C–C). Additionally, clear bands were identified at 2955.93 cm^−1^ and 2703.28 cm^−1^, corresponding to asymmetric and symmetric C–H stretching vibrations of the methylene groups, respectively. The intense peak observed at 1685 cm^−1^ was attributed to the presence of ester bonds, a characteristic feature of triglycerides within the oil sample [26].

In the analysis conducted by Dilara Yalmancı et al., the utilisation of FT-IR spectra was employed to elucidate the interactions between whey protein isolate and microbial exopolysaccharides within a low-fat sauce formulation. The obtained spectra demonstrate evident alterations in the region’s indicative of protein and carbohydrate functional groups, particularly at 1650 cm^−1^ (C=O bonds—amide I), 1540 cm^−1^ (amide II) and 3300–3400 cm^−1^ (hydroxyl and amine groups –O–H and N–H) [30].

In comparison, the FT-IR spectra obtained in this study demonstrate the presence of identical absorption regions in the sauce samples formulated with soy, pea and egg yolk proteins. However, variations in intensity and shape of the bands are observed, contingent on the protein source. These variations are indicative of specific interactions between proteins and the other components of the emulsion (water, oil, emulsifiers), reflecting the different chemical structure of each ingredient used. The two sets of spectra demonstrate notable similarities, which indicates that the essential functional interactions of the emulsified system can be monitored by FT-IR, irrespective of the protein source (animal or vegetable origin). This observation is particularly significant in regions that are sensitive to proteins and hydrophilic compounds.

### 3.3. Rheological Determinations

#### Steady State Rheology

**Figure 2:**
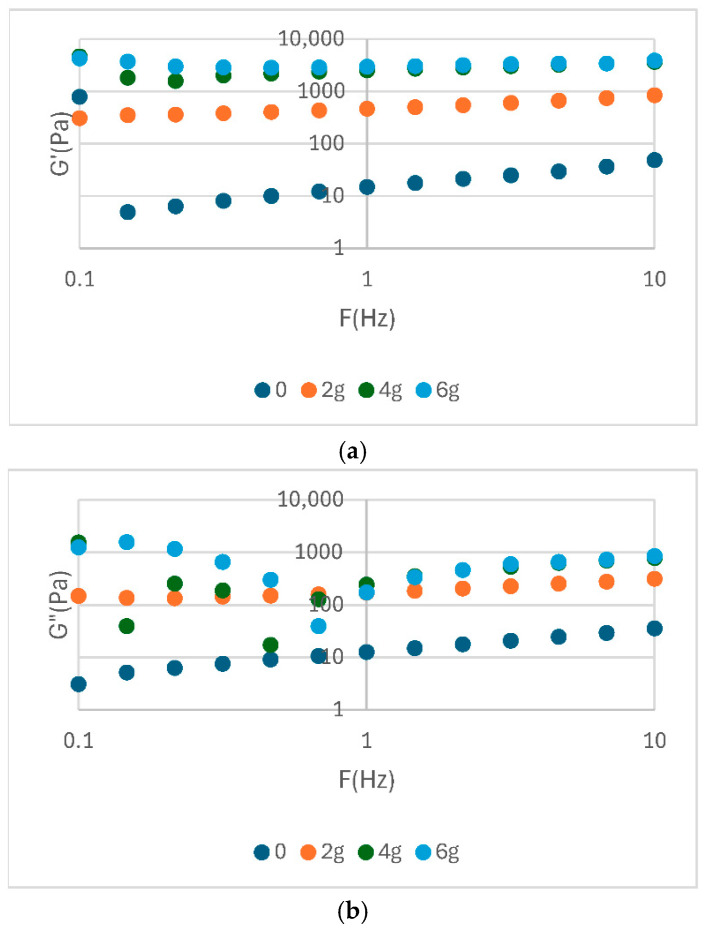

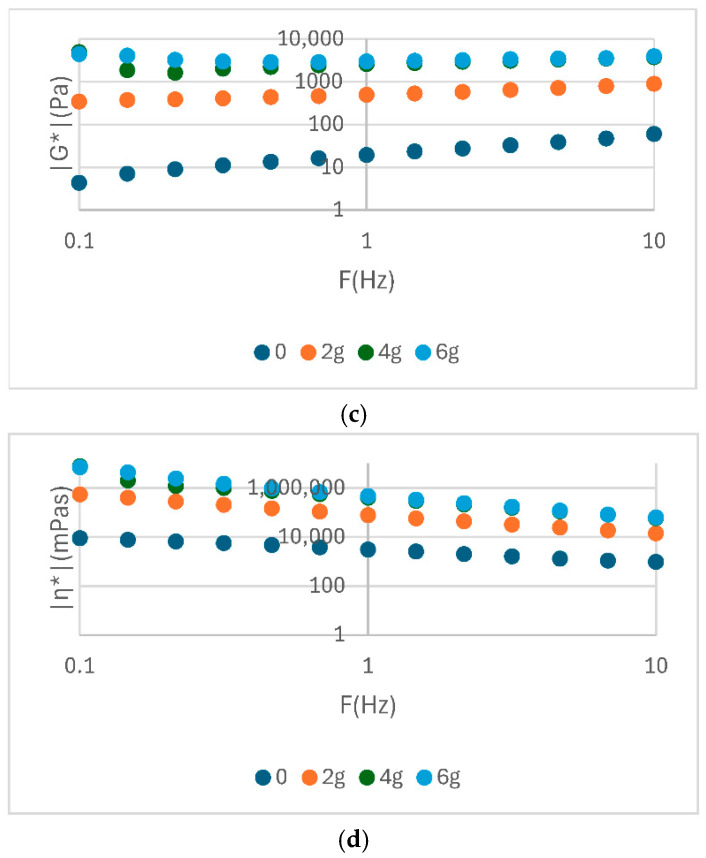
Viscoelastic properties of yolk-powder sauce samples as a function of frequency: (**a**) storage modulus G′; (**b**) loss modulus G″; (**c**) complex modulus |G*|; (**d**) complex viscosity |η*|.

**Figure 3:**
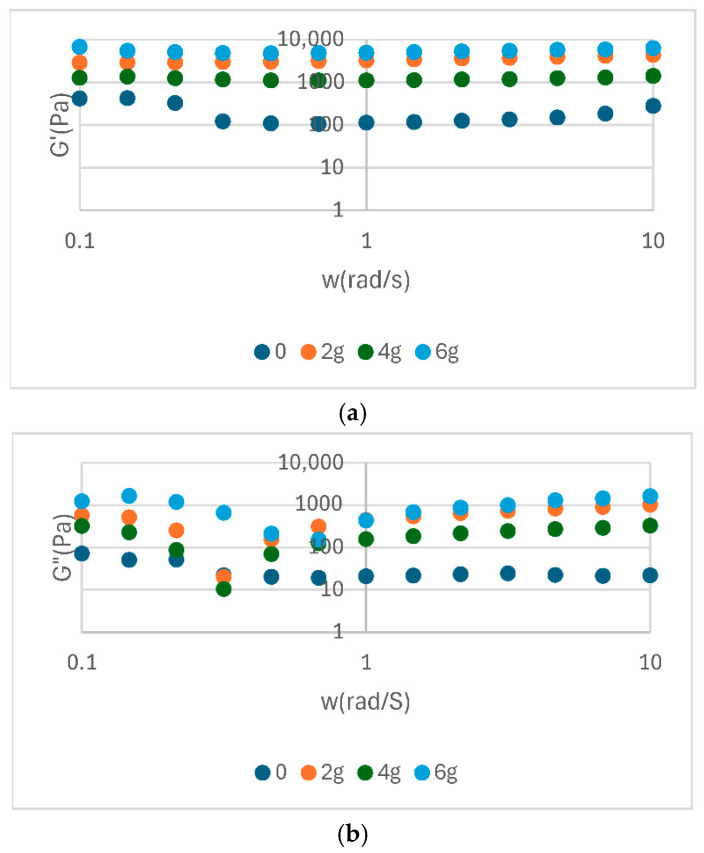

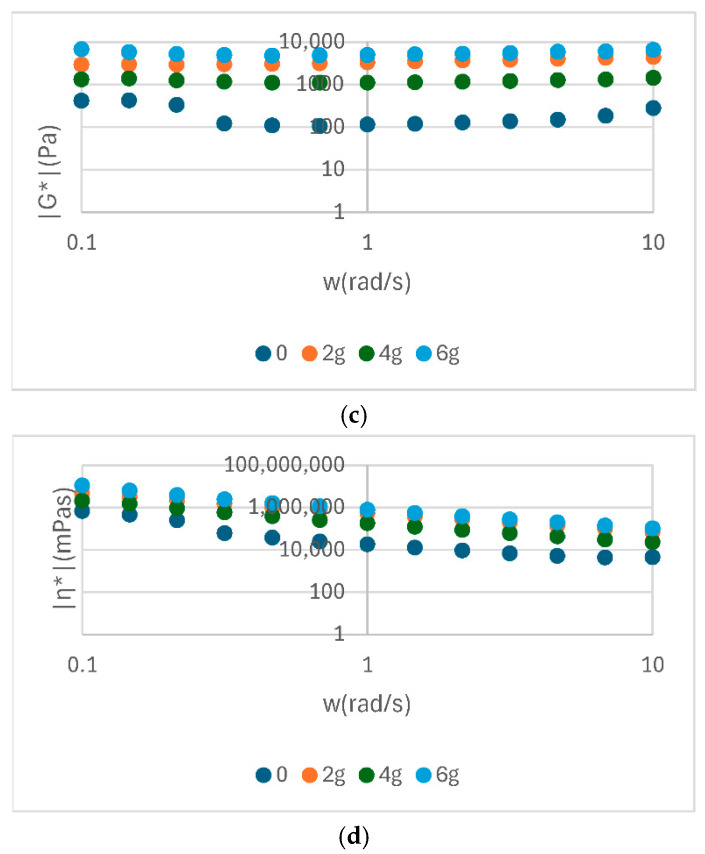
Viscoelastic properties of soy protein sauce samples as a function of frequency: (**a**) storage modulus G′; (**b**) loss modulus G″; (**c**) complex modulus |G*|; (**d**) complex viscosity |η*|.

**Figure 4:**
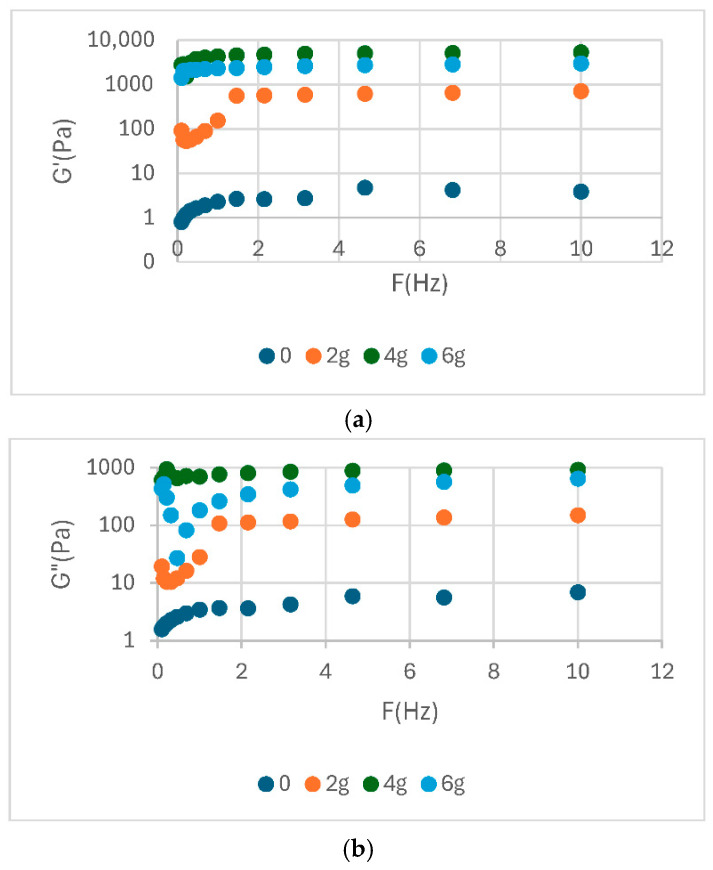

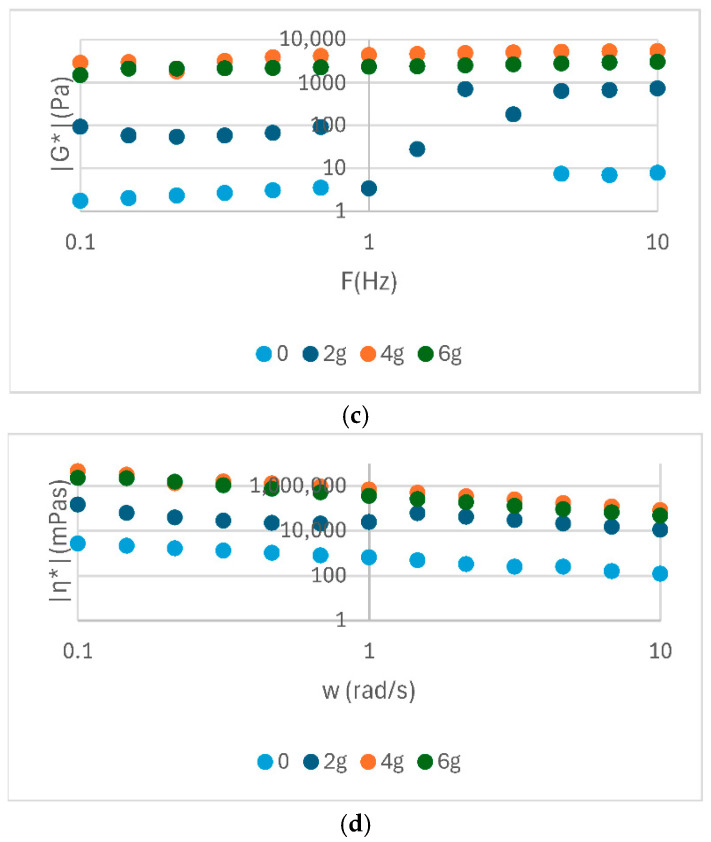
Viscoelastic properties of pea protein sauce samples as a function of frequency: (**a**) storage modulus G′; (**b**) loss modulus G″; (**c**) complex modulus |G*|; (**d**) complex viscosity |η*|.

The analysis of the rheological parameters presented in the table highlights significant differences between the analysed samples in terms of elastic modulus (G′), complex viscosity (|η*|) and complex modulus (|G*|) (Table 3). The rheological parameters are shows in Figure 2, Figure 3 and Figure 4. The elevated values of G′ and |G*| observed for samples S3 and P2 indicate a more stable structure and a more well-formed network, while low values, such as those exhibited by E0 and S0, suggest a weaker consistency. In addition, the η* values demonstrate a correlation with the observed flow behaviour, indicating an increased viscosity for samples with better organized structures.

**Figure 5:**
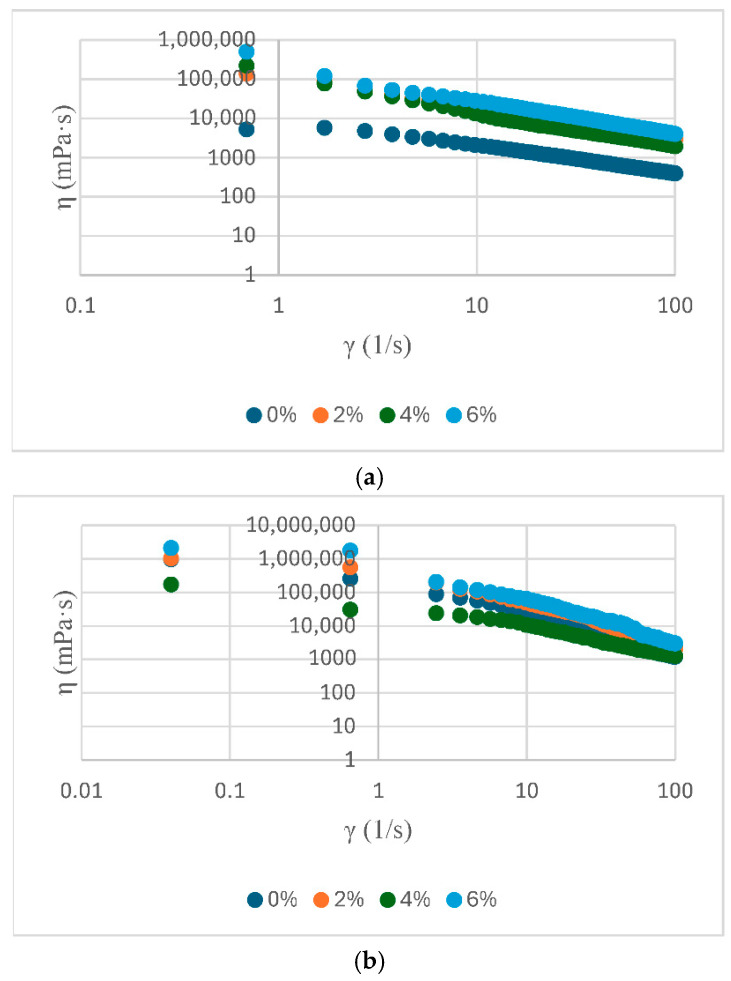

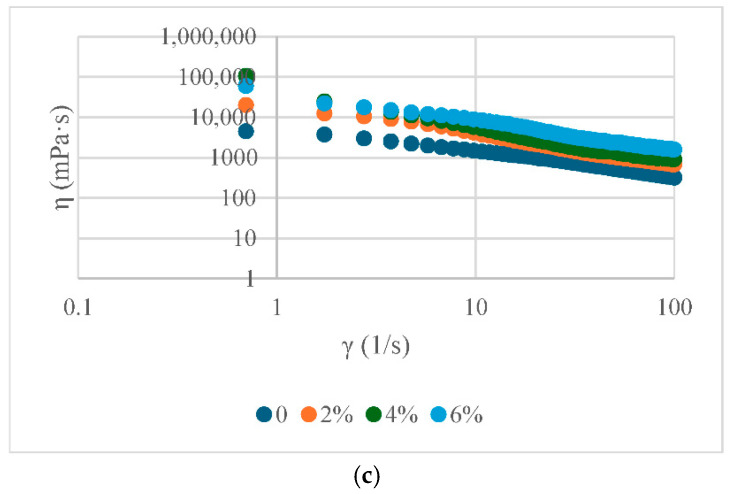
Dynamic viscosity for sauce samples: (**a**)—E, (**b**)—S, (**c**)—P.

In general, soy proteins (e.g., S3: E′ = 4908 Pa) exhibited a slightly higher elastic modulus than pea proteins (e.g., P2: G′ = 4330 Pa). Nevertheless, both showed high values of complex viscosity (|η*|), indicating optimal emulsification capacity and structural stability. The values presented in Table 3 provide quantitative support for the observations discussed in the previous diagrams. This provides a clear basis for confirming the differences in rheological behaviour between protein sources and treatments. The rheological behaviour of food emulsions, such as sauce, plays a crucial role in the perception of texture, the stability of the emulsion, and its sensory acceptability. In this study, a range of sauce samples were formulated with different protein sources (egg yolk powder, soy protein, and pea protein) at varying concentrations (0, 2, 4, and 6%) to evaluate their impact on flow properties (Figure 5).

The rheological properties of sauce were found to be significantly influenced by the type and concentration of protein additives utilised. This observation was supported by the results of the study, which showed that the apparent viscosity of the sauce changed noticeably with the applied shear rate. All three sets of samples exhibited distinct pseudoplastic behaviour, characterised by a decrease in viscosity with an increase in shear rate. This phenomenon is commonly observed in emulsified systems with complex structures. In samples enriched with yolk powder, the highest apparent viscosity was observed at low shear rates, especially for concentrations of 4% and 6%. This suggests the presence of a well-formed internal network and a high-water retention capacity, which are commonly associated with firmer texture and potentially improved emulsion stability in similar systems. As the rate of shear increases, the viscosity of the mixture decreases; however, the disparities in concentration remain discernible, thereby underscoring the pivotal structural function of the yolk components. For the samples formulated with soy protein, the initial viscosity is noticeably lower compared to those based on yolk, and the differences between concentrations are less pronounced. This phenomenon may indicate a less developed gelation network or a less efficient interaction between soy proteins and the oil phase. However, the pseudoplastic behaviour is maintained, suggesting that soy protein contributes to the stability of the system, albeit to a lesser extent. These lower viscosity values, particularly in soy- and pea-based samples, are consistent with expectations for reduced-fat emulsified systems and align with findings reported in the literature.

The pea protein samples exhibited elevated viscosity values at the initial stage of the curve, showing similarities to those of egg yolk powder, particularly at higher concentrations. The closeness of these curves suggests stable and predictable rheological behaviour. Pea protein appears to possess a satisfactory capacity for system structuring, with the capability to effectively substitute conventional animal-derived components. The rheological profile of the emulsions appeared to be influenced by the type of pro-tein source used within the context of the tested formulations. While egg yolk samples exhibited the most favorable viscoelastic properties, pea protein demonstrated stable and viscous behavior, suggesting its potential as a viable plant-based structuring agent. Soy protein provided intermediate characteristics. However, these observations must be interpreted with caution, as the samples also differed in fat content and the presence of tomato pomace, which may have contributed to the observed rheological behavior. Direct comparisons are therefore limited by the multifactorial differences between formulations.

In this study, the rheological behaviour of mayonnaise samples was compared with that reported by Raviteja Miriyala et al.; who analysed a standard commercial mayonnaise subjected to constant shear tests. Their study employed two mathematical models to describe the material’s behaviour: the viscoelastic-plastic (EVP) model and the two-element viscoelastic-plastic (TEVP) model. The utilisation of these models facilitated accurate simulation of the material’s response at varying shear rates, resulting in smooth and predictable curves [35].

The rheological behavior observed in the P2 formulation, which included pea protein and tomato pomace, indicates the formation of a well-developed structural network, as evidenced by its high elastic modulus (G′ = 4330 Pa) and complex viscosity (|η*| = 14,939 mPa·s). These values are comparable to those reported by Lee et al. for the formulation containing 3% defatted soybean flour, where the removal of oil components enhanced the emulsifying functionality of the soy matrix and contributed to improved structural integrity.

The observed pseudoplastic flow behavior, marked by a progressive decrease in viscosity with increasing shear rate, aligns with the typical viscoelastic nature of stable oil-in-water emulsions such as mayonnaise. From a microstructural perspective, our formulation yielded a relatively uniform distribution of oil droplets, although the average droplet size remained slightly larger compared to the formulation containing 3% egg yolk powder. A similar tendency was observed by Lee et al., where plant-based emulsions exhibited larger droplet sizes than their egg yolk counterparts, despite showing good dispersion [24].

These findings support the functional potential of pea protein combined with dietary fiber from tomato pomace as a viable plant-based structuring system in reduced-fat mayonnaise-type emulsions. However, the slightly coarser droplet size compared to egg yolk-based formulations may indicate a lower interfacial activity or stabilizing efficiency of pea protein. This underscores an important trade-off between achieving clean-label, plant-derived emulsions and replicating the fine structural characteristics of traditional mayonnaise. Further optimization of plant protein emulsifiers may therefore be necessary to fully match the rheological and microstructural performance of conventional, animal-based systems.

In comparison, the samples analysed in this study—formulated with egg yolk powder, soy protein, and pea protein—exhibited the same pseudoplastic behaviour, but with significant variations depending on the composition. For instance, the addition of egg yolk powder to sauce resulted in increased viscosity and shear strength, closely matching the predictions of the EVP model in the low shear rate region. Samples containing soy protein exhibited a reduction in viscosity, approaching with the theoretical behaviour predicted by TEVP model, particularly in the medium to high shear range. Samples containing pea protein exhibited a stable profile comparable to that of egg yolk, but with a more gradual decline in viscosity. In the study conducted by Xuelian Jing et al., it was established that the composition of mayonnaise can be modified by partially replacing fat with modified starch and dietary fibre (formulations named YFM Fifty, YFM Twenty, etc.). This modification exerts a direct influence on the size of the oil phase droplets in the emulsion. The findings demonstrated that a reduced mean droplet size (for example, 6.63 µm in the case of the YFM_30_ formulation) is correlated with a more compact and robust internal network, indicating enhanced rheological capabilities of the product [36].

The present study observed a similar trend, with sauce formulated using higher concentrations of egg yolk powder or vegetable proteins (4% and 6%, respectively) exhibiting higher viscosities at low shear rates, along with clearly defined pseudoplastic behaviour. This rheological response indicates the formation of a well-organised internal structure, likely supported by a finer and more uniform dispersion of oil droplets. This finding is consistent with the formulations obtained by Jing. It has been demonstrated that the droplet size exerts a direct influence on the rheological behaviour and emulsion stability.

Oscillatory rheological analysis of sauce with the addition of yolk powder shows a predominantly elastic behavior (G′ > G″), characteristic of well-structured systems. The modulus values increase with concentration, indicating a strengthening of the internal network. The tangent of the loss angle (tan δ < 1) confirms the solid-like character, and the complex viscosity decreases slightly with frequency, indicating structural stability. This, the addition of yolk powder enhances the firmness and stability of sauce, especially at higher concentrations (4% and 6%).

Rheological analysis of soy protein sauce shows a dominant elastic behaviour (G′ > G″), indicating a stable network. The moduli values increase with protein concentration, especially at 4% and 6%, suggesting a strengthening of the texture. The tangent of the loss angle remains below 1, indicating a solid-like system. The complex viscosity decreases slightly with frequency, reflecting a balanced and stable internal structure.

Rheological analysis of pea protein sauce shows a significant increase in the G′ and G″ moduli with increasing concentration, particularly at 6%, indicating a well-defined elastic structure. G′ remains higher than G″, confirming the dominant elastic behaviour. The tangent of the loss angle stays below 1, supporting the solid-like character of the system. The complex viscosity is high and stable, especially at high concentrations, suggesting dense, deformation-resistant network.

### 3.4. Microstructure of Sauce Samples

**Figure 6:**
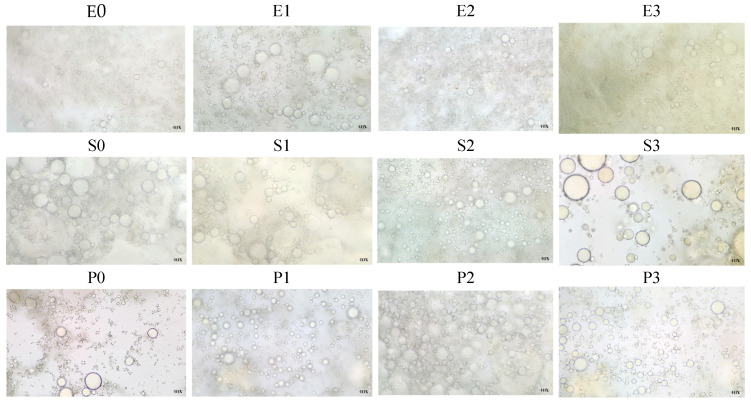
Sauce optical microstructure 40×. egg yolk-E, soy-S, or pea protein-P.

A microstructural analysis of sauce-type emulsions obtained with different emulsifiers and tomato pomace addition was conducted (Figure 6). In this study, a microstructural analysis was performed on sauce samples formulated with different emulsifiers: egg yolk powder (serving as the animal-based control), soy protein, and pea protein (as plant-based alternatives). Additionally, the impact of tomato pomace supplementation (2, 4, and 6 g) on emulsion structure was investigated, from the perspectives of physico-chemical stability and the enhancement of nutritional value. The product in question is based egg yolk and is commonly referred to as sauce. The control sample exhibited a microstructure indicative of a stable emulsion, characterised by finely dispersed, minute oil droplets, confirming the effectiveness of yolk lecithin as an emulsifier. The incorporation of tomato pomace resulted in a gradual increase in oil droplet size. At a concentration of 6 g, visible aggregates and larger lipophilic structures were formed, indicating a tendency toward coalescence and potential emulsion destabilization. Nonetheless, the presence of pomace caused a slight reddening of the colour, indicating potential sensory enhancement and the presence of natural antioxidants.

The emulsion obtained exclusively with soy protein (the plant-based control) presented a heterogeneous microstructure with larger oil globules, indicating a lower emulsification capacity in comparison to the yolk. The incorporation of tomato pomace at concentrations of 2 and 4 g led to a noticeable improvement in the structure, characterised by more uniform droplet distributions and reduced droplet size. This observation suggests a potential stabilising effect of the phenolic compounds and soluble fibres present in the tomato pomace. However, at a concentration of 6 g, the emulsion exhibited signs of instability, characterised by a propensity for aggregation and partial phase separation. Such variations in viscosity and emulsion stability cannot be attributed solely to the protein type or pomace level, but rather to their interaction, as evidenced by consistent patterns across multiple analytical parameters. These microstructural observations align with the trends observed in viscosity and phase separation analyses, suggesting that formulations with moderate tomato pomace and soy protein achieved the best balance between droplet dispersion and structural stability. The pea protein control sample exhibited the least developed microstructure among the three samples, characterised by the presence of large, fused oil globules and uneven distribution. At moderate pomace concentrations (2 and 4 g), a slight improvement in droplet dispersion was observed, though not to the same extent as with soy protein. As the concentration increased to 6 g, a pronounced destabilisation of the structure was observed, accompanied by the emergence of dense aggregates and distinct zones of phase separation.

The replacement of egg yolk with plant-based proteins is technologically feasible, particularly when soy protein is used. Additionally, the incorporation of tomato pomace has been shown to enhance both the structural integrity and nutritional value of the product. However, it is important to exercise caution and utilise this additive in moderation to prevent the disruption of the emulsion stability.

While the present microstructural evaluation was based on qualitative observation, future studies could benefit from quantitative image analysis to measure droplet size distribution and aggregation patterns. The availability of such data would facilitate a more objective comparison between emulsifier types and the impact of tomato pomace concentration on emulsion stability.

These patterns align with prior findings by Jing et al., who used CLSM imaging to evaluate mayonnaise prepared with pea protein isolate (PPI) and PPI–xanthan gum conjugates. Their study demonstrated that improved surface coverage and finer oil droplet dispersion correlated with enhanced emulsion stability, especially in conjugate-based formulations. Furthermore, as xanthan gum concentration increased, a denser network structure was observed, indicating that protein–polysaccharide conjugation may significantly improve emulsion performance and plasticity [36].

In a recent study, Akhtar and Masoodi investigated the effects of incorporating high-quality vegetable oil, both in free form and microencapsulated in matrices based on isolated soy protein, maltodextrin and, pectin, on the microstructure of mayonnaise. Optical microscopy observations revealed that the free-form control sample, containing high-quality vegetable oil without the polysaccharide-protein complex, presented significantly larger oil droplets with a tendency to coagulate, indicating low emulsion stability. Mayonnaise samples enriched with Pickering emulsions stabilised by isolated soy, maltodextrin, and pectin exhibited a well-dispersed oil-in-water structure, characterised by spherical, small, and homogeneous oil droplets. The incorporation of soy protein isolate and pectin enhanced stabilisation of the oil-water interface and reduce coalescence. Furthermore, lowering the pH near the isoelectric point of soy protein isolate has been shown to promote electrostatic interactions with pectin, thereby facilitating the formation of a stable network within the emulsion [13].

### 3.5. Analysis of Organoleptic Properties

The sensory evaluation of sauce samples was conducted using a 9-point hedonic scale, ranging from 1 (“dislike extremely”) to 9 (“like extremely”). A panel of 20 semi-trained assessors, aged between 18 and 65, evaluated each of the 12 samples under standardised conditions. The assessed attributes included colour, odour profile, taste, creaminess, consistency, overall appearance, overall acceptability, as well as an average score calculated as the arithmetic mean of all criteria. Each panelist evaluated all the criteria, and the mean scores were then calculated. No recalibration or curve fitting was applied; raw scores were used as collected.

**Figure 7:**
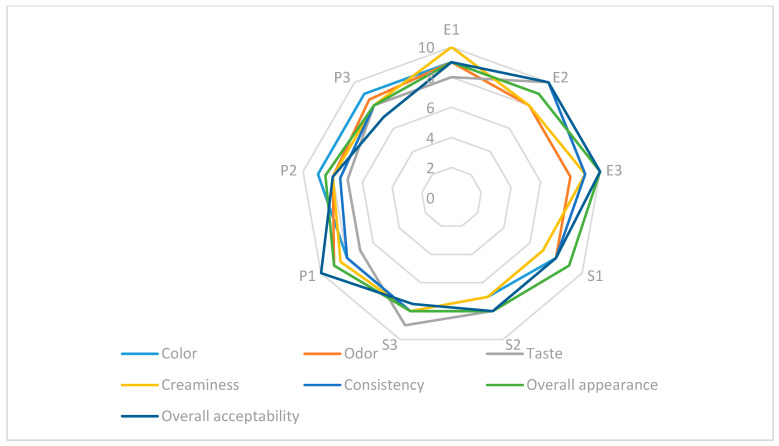
Sensory evaluation of sauce samples based on eight criteria. egg yolk-E, soy-S, or pea protein-P.

The sensory results, visualised in the radar plot in Figure 7, revealed clear differences between the formulations. Samples based on egg yolk (E0–E3) received the highest scores for the most attributes, particularly taste, creaminess, and overall acceptability. This finding confirms the superior sensory performance of conventional recipes. The sensory evaluation of the soy protein samples (S0–S3) revealed intermediate scores, with satisfactory ratings for consistency and appearance ratings but lower ratings for odor and taste.

Samples formulated with pea protein (P0–P3) generally scored lower across all sensory parameters, especially in terms of taste and odor, indicating limited consumer acceptability in their current form. However, among these, sample P2 demonstrated a relatively higher score in consistency, suggesting functional potential in terms of texture, even though its overall hedonic appeal remained modest. Consequently, while P2 is not considered optimal in sensory acceptability, it may serve as a technologically promising foundation for future reformulations aimed at improving flavour and aroma profiles. These findings emphasise that plant-based formulations may offer structural functionality but require significant sensory optimisation to meet consumer expectations.

In the study conducted by Jorge Metri-Ojeda et al., the sensory evaluation of low-fat mayonnaises revealed that factors such as taste, texture, and overall acceptability were significantly influenced by product composition, particularly protein and fat content. The findings indicate that formulations with an optimal balance consistently achieved the highest sensory ratings. This conclusion is further supported by the correlation matrix presented in the paper [25]. Similarly, in the sensory evaluation carried out in this study, the highest scores were obtained by sauce with yolk powder, especially for attributes such as taste, creaminess, and overall acceptability. Samples with pea protein had intermediate scores, while those with soy protein recorded lower ratings. Thus, as in the research of Jorge Metri-Ojeda et al., the importance of composition in the sensory perception of mayonnaise is confirmed. In a recent study by Mohammadi et al., a low-fat mayonnaise formulation was developed in which egg yolk was partially replaced with amaranth protein isolate (API). The results demonstrated that, at optimal substitution levels, the samples maintained acceptable sensory characteristics, particularly in terms of taste, texture, and overall appearance, with no significant differences compared to the control formulation. These findings align with the results obtained in the present study, where egg yolk powder-based sauces received the highest sensory scores for taste, creaminess, and overall acceptability [37]. In contrast, the pea protein-based samples (P0–P3) in this study showed lower scores, especially for taste and odor, indicating limited consumer acceptability in their current form. When compared to the API-based formulations proposed by Mohammadi et al., the pea protein samples appear less favorable in sensory integration. This comparison reinforces the idea that the type and functional quality of the protein used as a yolk replacer has a direct impact on sensory perception. It also emphasizes the importance of careful selection and optimization of plant-based ingredients when developing low-fat sauces intended to meet consumer expectations.

### 3.6. Principal Component Analysis

**Figure 8:**
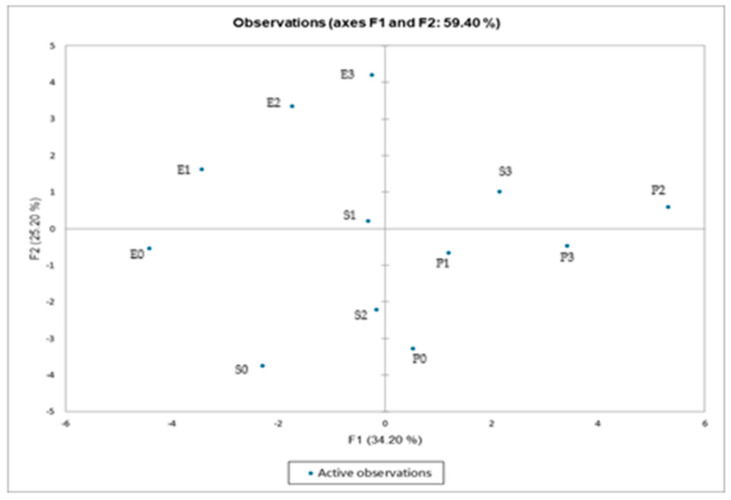
PCA score plot showing the distribution of oil extract samples (●) on the F1 × F2 factorial plane (59.40% of total variance). Clustering reveals grouping patterns related to type of oil and treatment applied.

**Figure 9:**
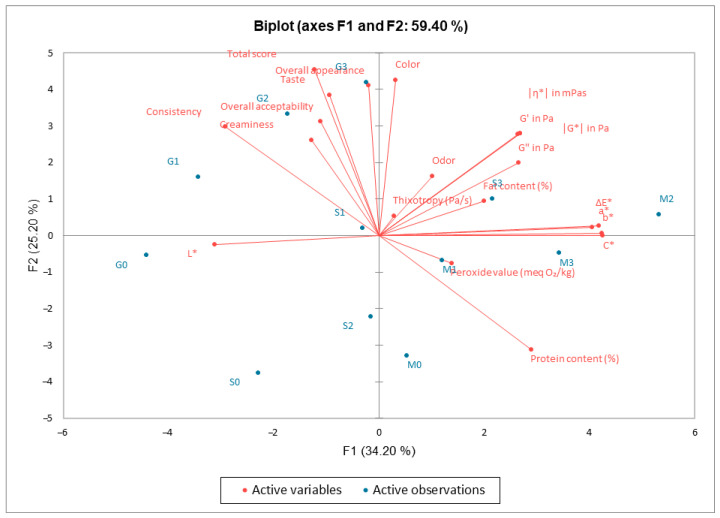
PCA biplot illustrating the correlation between sample scores (●) and variable loadings (→), including color parameters (L*, a*, b*, C*, and ΔE*), physicochemical properties (fat content, protein content, and peroxide value), and sensory descriptors (odor, taste, color, consistency, and overall acceptability).

Figure 8 and Figure 9 present the principal component analysis. As shown in Figure 8, the distribution of samples is depicted along the first two principal components (F1—34.2%, F2—25.2%), which together account for 59.4% of the total data variability. The samples are grouped according to shared characteristics. For example, E2, E3, and S1 are proximate, indicating similar profiles. It is evident that samples P2 and P3 are distinctly separated along the F1 axis, suggesting a divergent behaviour relative to the other samples. Furthermore, E0 and S0 are positioned away from the others, suggesting the presence of distinct features. Figure 9 presents a simultaneous representation of the samples and the variables that contributed to the formation of the principal components. The variables are represented as vectors (red arrows), where their direction and length indicate their influence on the components. Samples located near a variable exhibit a high value for that characteristic. For instance, P2 shows a strong correlation with colour parameters (b*, ΔE*, and C*) and protein content. The variables “Taste”, “Appearance”, and “Overall Acceptability” were found to be positively correlated, indicating that sensory ratings were aligned. The presence of opposite vectors indicates a negative relationship. For example, the colour “L*” appears to be diametrically opposed to positive sensory qualities, suggesting that a lighter colour is associated with lower scores. The study demonstrated that sauce samples based on egg yolk (E0–E3) exhibited superior sensory and rheological properties compared to formulations based on soy (S0–S3) and pea proteins (P0–P3). The conventional samples received the highest ratings in terms of taste, creaminess, and overall acceptability, thereby confirming their well-established sensory appeal. Soy protein was found to be a viable alternative, providing acceptable texture and structure, although improvements in flavour and aroma are required. Pea protein formulations demonstrated the lowest overall sensory scores, particularly in terms of taste and odour. However, certain samples (e.g., P2) exhibited good consistency, suggesting potential for optimisation. From a rheological perspective, both soy and pea proteins contributed to the formation of structured emulsions, with variations in viscosity and viscoelastic properties depending on concentration and formulation. While plant-based proteins offer a promising alternative for developing egg-free sauce, further formulation adjustments and ingredient synergies are necessary to improve their sensory profile and better meet consumer expectations. These findings are consistent with those reported by Motawee et al., who developed low-fat mayonnaise formulations using quinoa protein isolate and xanthan gum. In their study, the quinoa-based samples demonstrated acceptable rheological and structural properties, similar to those observed with soy and pea proteins in the present work. However, quinoa-based samples also received lower scores for taste and aroma compared to egg-based controls, highlighting the sensory limitations commonly associated with plant proteins. Their PCA analysis confirmed that sensory and compositional variables were closely correlated, with clear grouping patterns according to protein type and formulation—further reinforcing the conclusion that taste and overall acceptability are strongly influenced by ingredient functionality. As with soy and pea protein formulations, the study by Motawee et al. demonstrates that plant-based alternatives require targeted improvements to compete with conventional egg-based emulsions in sensory quality [38].

## 4. Limitation and Future Direction

Nevertheless, several limitations should be acknowledged. First, the experimental design focused on a limited set of emulsifiers and tomato pomace concentrations, which may not capture the full spectrum of interactions or optimal formulation ranges. Second, the microstructural evaluation relied primarily on qualitative microscopy, without quantitative image analysis for droplet size distribution or aggregation indices. Finally, while comparisons were made with mayonnaise literature, the differences in ingredient ratios and processing steps may limit the direct transferability of some conclusions. These aspects warrant further investigation in future research.

In addition, the method employed for fat content determination—direct Soxhlet extraction on fresh emulsified samples—represents a methodological limitation. While this approach aimed to preserve the native emulsion structure, the absence of prior dehydration or acid hydrolysis may have resulted in underestimated fat values due to the high water content of the samples. This factor should be taken into account when interpreting fat-related comparisons across formulations. Future studies are encouraged to adopt improved extraction protocols, such as sample drying or hydrolysis-assisted Soxhlet methods, to enhance the accuracy of lipid quantification in high-moisture emulsions. Future research could explore the use of additional plant-based proteins—such as lentil, chickpea, or rice protein—as alternative emulsifiers, aiming to optimise emulsion stability, improve nutritional profiles, and support the development of clean-label, vegan products. Simultaneously, further investigations may focus on optimising both the concentration and pre-treatment of tomato pomace to enhance its antioxidant capacity and stabilising effects without compromising sensory quality. The promising functionality of these formulations also suggests the potential for scale-up in industrial production, where process refinement, shelf-life validation, and regulatory compliance would be key considerations. Beyond sauces, the incorporation of tomato pomace could be extended to a wider range of food matrices, including bakery items, plant-based spreads, and meat analogues, thereby supporting circular economy principles and contributing to the creation of fibre-rich, sustainable foods.

## 5. Conclusions

The study emphasised the impact of different protein types on the compositional, sensory, and functional characteristics of sauce. PCA analysis demonstrated a clear separation among samples P0–P3, indicating that the nature of the protein significantly contributes to variations in product quality. The utilisation of Fourier-transform infrared spectroscopy (FT-IR) as a diagnostic tool confirmed the presence of specific protein-lipid interactions unique to each protein type. The resulting spectra suggested potential structural differences, which may consequently influence rheological properties and stability of the sauce. Regarding colour, significant variations were observed among the samples, particularly in the a*, b*, C*, and ΔE* values. The P2 sample exhibited the most pronounced colour shades, a crucial attribute for the product’s aesthetic appeal. Rheological analysis revealed significant differences in the viscoelastic behaviour of the samples. As demonstrated in Figure 1, sample P2 exhibited elevated values for G′, G″, and thixotropy, suggesting a more robust and stable structure, which is optimal to produce a high-quality sauce. Finally, the sensory evaluation corroborated these instrumental analyses: P2 was perceived as having the best consistency, taste, and overall acceptability, followed by P3. Therefore, sample P2 emerges as a promising prototype for the development of clean-label emulsions based on plant-derived proteins, particularly in applications targeting improved texture, nutritional quality, and consumer appeal. However, signs of microstructural instability at higher pomace concentrations in P2 were previously reported, raising concerns about long-term emulsion stability. This highlights the need for additional formulation tuning—such as protein–polysaccharide conjugation or incorporation of stabilisers—to maintain physical integrity under fibre-rich conditions. Furthermore, due to the simultaneous variation of multiple formulation parameters (protein type, oil-to-water ratio, pomace concentration), the observed effects reflect combined rather than isolated contributions. This experimental limitation should be considered when interpreting the results. Future designs should decouple individual variables to strengthen causal interpretations and identify optimal ingredient interactions.

Overall, this study contributes to the development of sustainable and nutritionally enriched emulsified products by valorising tomato pomace and exploring alternative proteins. Continued research should focus on process optimisation, shelf-life extension, and consumer acceptance to support industrial translation of these formulations. In conclusion, although P2 shows promising characteristics, future research should aim to decouple individual factor effects through more controlled designs and explore stabilisation strategies to address structural weaknesses, thus enhancing formulation robustness and ensuring greater reproducibility.

## Figures and Tables

**Table 1:** Amount of ingredients used to prepare the sauce.

Ingredients (%)	E0	E1	E2	E3	S0	S1	S2	S3	P0	P1	P2	P3
E/S/P	25.4	18.4	15.8	13.9	12.0	11.7	10.6	9.7	14.2	10.9	9.4	9.1
Water	47.6	46.0	49.5	52.2	60.2	65.7	66.2	66.7	61.9	54.4	58.5	68.6
Pomace of tomatoes	0.0	4.6	7.9	10.4	0.0	2.9	5.3	7.3	0.0	2.7	4.7	6.9
Vinegar	3.2	2.3	2.0	1.7	1.5	1.5	1.3	1.2	1.8	1.4	1.2	1.1
Sunflower oil	15.9	23.0	19.8	17.4	22.6	14.6	13.2	12.1	17.7	27.2	23.4	11.4
Mustard	6.3	4.6	4.0	3.5	3.0	2.9	2.6	2.4	3.5	2.7	2.3	2.3
Salt	1.6	1.1	1.0	0.9	0.8	0.7	0.7	0.6	0.9	0.7	0.6	0.6

egg yolk-E, soy-S, or pea protein-P.

**Table 2:** Physico-chemical properties of the analyzed the sauce samples.

Sample	Peroxide Value (Meq O_2_/kg)	Fat Content (%)	Protein Content (%)	L*	a*	b*	C*	Δ E *
E0	1.67 ^a^ ± 0.04	32.37 ^c^ ± 0.04	10.44 ^c^ ± 0.05	67.53 ^b^ ± 0.15	8.10 ^a^ ± 0.11	30.27 ^a^ ± 0.13	31.08 ^a^ ± 0.06	0
E1	2.20 ^a^ ± 0.1	34.9 ^d^ ± 0.05	7.58 ^b^ ± 0.05	68.53 ^c^ ± 0.15	8.80 ^b^ ± 0.12	31.87 ^b^ ± 0.1	33.08 ^b^ ± 0.01	2.01 ^b^ ± 0.15
E2	2.80 ^a^ ± 0.05	30.10 ^b^ ± 0.2	6.55 ^a^ ± 0.05	68.99 ^d^ ± 0.05	17.17 ^c^ ± 0.10	36.25 ^c^ ± 0.8	40.12 ^c^ ± 0.10	10.96 ^c^ ± 0.10
E3	3.02 ^a^ ± 0.04	26.43 ^a^ ± 0.15	5.77 ^a^ ± 0.04	57.27 ^a^ ± 0.08	17.58 ^c^ ± 0.17	37.54 ^d^ ± 0.1	41.45 ^d^ ± 0.16	15.74 ^d^ ± 0.09
*F*-value	1.19 ns	548.40 *** ± 0.05	284.19 *** ± 0.05	11,155.52 *** ± 0.04	3918.90 *** ± 0.3	719.25 *** ± 0.2	1412.19 *** ± 0.06	4783.56 *** ± 0.08
S0	3.75 ^d^ ± 0.15	23.16 ^d^± 0.15	4.94 ^c^ ± 0.05	69.57 ^c^ ± 0.18	13.29 ^a^ ± 0.08	32.13 ^a^ ± 0.14	35.69 ^a^ ± 0.16	0
S1	2.04 ^a^ ± 0.14	15.18 ^c^ ± 0.05	4.81 ^b^ ± 0.08	70.57 ^d^ ± 0.14	15.29 ^b^ ± 0.06	35.33 ^b^ ± 0.11	38.49 ^b^ ± 0.13	9.30 ^b^ ± 0.1
S2	2.27 ^b^ ± 0.05	13.77 ^b^ ± 0.05	4.3 ^b^ ± 0.05	62.49 ^a^ ± 0.09	15.54 ^b^ ± 0.16	38.3 ^c^ ± 0.15	41.33 ^c^ ± 0.1	11.91 ^c^ ± 0.08
S3	2.67 ^c^ ± 0.02	12.61 ^a^ ± 0.05	4.02 ^a^ ± 0.09	65.62 ^b^ ± 0.13	20.6 ^c^ ± 0.15	41.4 ^d^ ± 0.05	46.24 ^d^ ± 0.14	16.84 ^d^ ± 0.03
*F*-value	228.92 *** ± 0.14	926.33 *** ± 0.10	28.43 *** ± 0.05	4157.87 *** ± 0.14	2655.31 *** ± 0.1	4617.68 *** ± 0.16	6172.34 *** ± 0.2	1357.67 *** ± 0.11
P0	3.02 ^b^ ± 0.03	18.41 ^b^ ± 005	5.82 ^c^ ± 0.10	60.12 ^b^ ± 0.11	19.11 ^a^ ± 0.12	40.25 ^a^ ± 0.07	45.19 ^a^ ± 0.08	0
P1	5.27 ^d^ ± 0.05	27.76 ^d^ ± 0.1	4.49 ^b^ ± 0.05	61.12 ^c^ ± 0.11	19.77 ^b^ ± 0.09	41.75 ^b^ ± 0.11	46.19 ^b^ ± 0.1	17.13 ^b^ ± 0.11
P2	3.47 ^c^ ± 0.10	23.86 ^c^ ± 0.05	3.87 ^a^ ± 0.13	57.24 ^a^ ± 0.07	24.65 ^c^ ± 0.15	48.79 ^c^ ± 0.02	54.67 ^d^ ± 0.17	26.88 ^c^ ± 0.18
P3	2.20 ^a^ ± 0.05	11.89 ^a^ ± 0.2	3.79 ^a^ ± 0.14	56.98 ^a^ ± 0.10	24.48 ^c^ ± 0.07	48.48 ^c^ ± 0.08	54.31 ^c^ ± 0.09	26.66 ^c^ ± 0.15
*F*-value	670.36 *** ± 0.05	2188.46 *** ± 0.12	55.71 *** ± 0.5	1315.94 *** ± 0.08	1279.06 *** ± 0.14	3276.69 *** ± 0.12	7607.66 *** ± 0.17	14,323.05 *** ± 0.08

Letters indicate significant differences between samples (*p* < 0.0001); ns—not significant (*p* > 0.05); ***—*p* < 0.0001; egg yolk-E, soy-S, or pea protein-P. The colorimetric parameters assessed included L* (lightness), a* (red-green component), b* (yellow-blue component), C* (chroma, or colour saturation), and ΔE* (total color difference).

**Table 3:** The analysis of the rheological parameters.

Sample	G′ (Pa)	G″ (Pa)	|G*| (Pa)	|η*| (mPas)	Hystereis Area (Pa/s)
E0	14.84 ± 0.15	12.71 ± 0.10	19.54 ± 0.10	3110 ± 20.00	742.5 ± 7.50
E1	464.3 ± 2.01	170.8 ± 1.01	494.7 ± 2.50	78,730 ± 517.33	4044 ±40.07
E2	2553 ± 7.51	245 ± 5.00	2564 ± 5.03	408,100 ± 2000.83	3268 ± 51.07
E3	2944 ± 7.55	172.8 ± 2.51	2949 ± 12.53	469,300 ± 2007.49	6190 ± 75.50
S0	112.9 ± 0.75	20.71 ± 0.25	114.8 ± 0.25	18,270 ± 150.44	10,480 ± 151.0
S1	3268 ± 15.10	443.3 ± 2.54	3298 ± 15.10	524,800 ± 3579.57	14,520 ± 151.0
S2	1106 ± 5.03	157.6 ± 2.50	1117 ± 7.51	177,800 ± 1509.97	3446 ± 50.05
S3	4908 ± 10.07	433 ± 2.52	4927 ± 7.51	784,100 ± 1517.67	23,950 ± 75.06
P0	2.291 ± 0.03	3.446 ± 0.10	3.446 ± 0.10	658.6 ± 12.52	797.5 ± 12.50
P1	156.1 ± 1.00	28.06 ± 0.50	3.446 ± 0.60	25,240 ± 300.89	1437 ± 35.02
P2	4330 ± 10.00	701.2 ± 10.02	4387 ± 20.40	698,200 ± 3579.36	1493 ± 35.05
P3	2314 ± 10.26	182.1 ± 2.51	2321 ± 15.39	369,400 ± 3538.36	1772 ± 40.60

egg yolk-E, soy-S, or pea protein-P.

## Data Availability

The original contributions presented in the study are included in the article, further inquiries can be directed to the corresponding author.
